# Summer Instruction

**Published:** 1850-06

**Authors:** 


SUMMER INSTRUCTION.

We invite attention to the card, on the cover of this Journal, of Drs. David W. Yandell & R. J. Breckenridge. These gentlemen offer an excellent opportunity to students, to enjoy bed-side clinics, and we advise those desirous of this species of improvement in sound medical knowledge, to commence with the beginning of the course in July.

